# Lithium promotes DNA stability and survival of ischemic retinal neurocytes by upregulating DNA ligase IV

**DOI:** 10.1038/cddis.2016.341

**Published:** 2016-11-17

**Authors:** Ying Yang, Nandan Wu, Sijia Tian, Fan Li, Huan Hu, Pei Chen, Xiaoxiao Cai, Lijun Xu, Jing Zhang, Zhao Chen, Jian Ge, Keming Yu, Jing Zhuang

**Affiliations:** 1 State Key Laboratory of Ophthalmology, Zhongshan Ophthalmic Center, Sun Yat-sen University, Guangzhou, China

## Abstract

Neurons display genomic fragility and show fragmented DNA in pathological degeneration. A failure to repair DNA breaks may result in cell death or apoptosis. Lithium protects retinal neurocytes following nutrient deprivation or partial nerve crush, but the underlying mechanisms are not well defined. Here we demonstrate that pretreatment with lithium protects retinal neurocytes from ischemia-induced damage and enhances light response in rat retina following ischemia–reperfusion injury. Moreover, we found that DNA nonhomologous end-joining (NHEJ) repair is implicated in this process because in ischemic retinal neurocytes, lithium significantly reduces the number of *γ*-H2AX foci (well-characterized markers of DNA double-strand breaks *in situ*) and increases the DNA ligase IV expression level. Furthermore, we also demonstrate that nuclear respiratory factor 1 (Nrf-1) and phosphorylated cyclic AMP-response element binding protein-1 (P-CREB1) bind to ligase IV promoter to cause upregulation of ligase IV in neurocytes. The ischemic upregulation of Nrf-1 and lithium-induced increase of P-CREB1 cooperate to promote transcription of ligase IV. Short hairpin RNAs against Nrf-1 and CREB1 could significantly inhibit the increase in promoter activity and expression of ligase IV observed in the control oligos following lithium treatment in retinal neurocytes. More importantly, ischemic stimulation triggers the expression of ligase IV. Taken together, our results thus reveal a novel mechanism that lithium offers neuroprotection from ischemia-induced damage by enhancing DNA NHEJ repair.

Retinal ischemia is linked to various disorders such as diabetic retinopathy, glaucoma and other retinopathies. For example, with primary glaucoma, the elevation of intraocular pressure (IOP) often results in ischemia that leads to a lack of blood being supplied to the retina, which decreases the supply of oxygen, imbalances ion transport, triggers oxidative stress and finally causes the death of retinal cells.^1, 2^ Primary neurons that have suffered ischemia display genomic fragility and show fragmented DNA, both *in vitro* and *in vivo*.^3, 4, 5, 6^ Moreover, improper repair or a failure to repair DNA breaks may lead to a 'domino effect', causing gene deletions, translocations and missegregation of large chromosome fragments, which may result in cell death or apoptosis.

DNA double-strand breaks (DSBs) repair includes homologous recombination (HR) and nonhomologous end-joining (NHEJ),^7^ and NHEJ is the predominant pathway for repair of DNA DSBs in postmitosis cells.^8^ At present, the reasons why DNA repair fails in ischemic neurocytes remains obscure, but hypothetical support of failed NHEJ may be a therapeutic approach for the treatment of retinal ischemic-induced damage.

Clinically, lithium has been used to treat bipolar disorder for more than 60 years. More recently, much evidence from both *in vitro* and *in vivo* experimental studies, as well as clinical analyses, demonstrated the neuroprotective effects of lithium against a variety of insults, including glutamate-induced excitotoxicity, ischemia-induced neuronal damage, radiation-induced brain injury and neurodegenerative conditions.^9^ Our previous study and the other groups also demonstrated *in vitro* that lithium protects retinal neurocytes against nutrient deprivation-induced apoptosis.^10, 11^ Lithium has emerged as a neuroprotective agent efficacious in preventing apoptosis-dependent cellular death.

The exact mechanisms underlying the neuroprotective effects of lithium are not well defined, but, currently, are thought to be mediated by more than one pathway: transcriptional regulation, inhibition of apoptosis and changes in neurogenesis.^12, 13, 14^ For example, in cultured neural cells and distinct areas of the brain, lithium increases the activity of two prominent transcription factors: activator protein-1 and phosphorylated cyclic AMP-response element binding protein-1 (P-CREB1).^15^ Lithium inhibition of GSK-3 also regulates the transcription and expression of factors critically involved in neuroprotective, neurotrophic, anti-inflammatory and neurogenic effects.^16, 17, 18^ Moreover, lithium inhibits cell apoptosis by regulating proapoptotic p53, Bax, caspase, HSP70 and cytochrome *c* release.^19, 20, 21^

In particular, pioneering studies noted that, during hypoxic–ischemic brain injury, lithium confers long-term protection against DNA damage.^20, 22^ It was revealed that lithium treatment protected irradiated hippocampal neurons from apoptosis and improved cognitive performance of irradiated mice.^23, 24^ Lithium reduced neural progenitor apoptosis in the hippocampus and ameliorated functional deficits after irradiation to the immature mouse brain.^13^ However, the mechanism of DNA repair that lithium targets when it ameliorates neural degeneration is unknown.

Using cultures of primary rat retinal neurocytes and ischemia–reperfusion (I/R) surgery model, we sought to determine whether pretreatment of lithium could protect retinal neurocytes in both *in vitro* and *in vivo* conditions, and we examined the possible underlying mechanisms, focusing on DNA NHEJ repair.

## Results

### Lithium increases DNA NHEJ repair and viability of retinal neurocytes after serum deprivation *in vitro*

**Figure 1:**
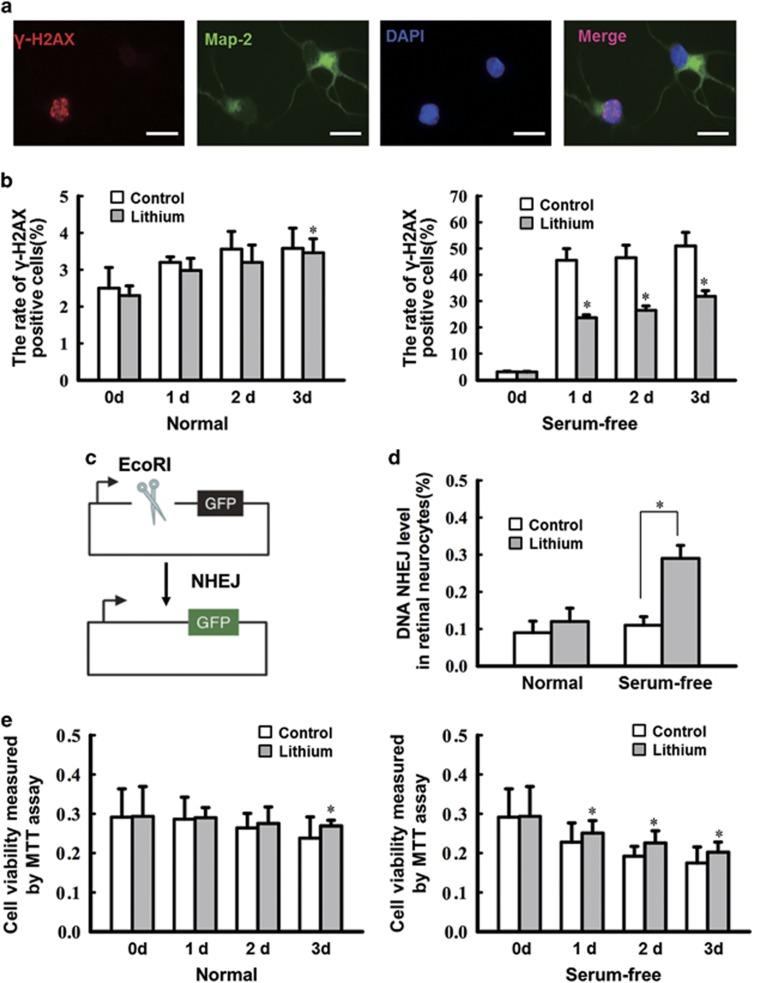
Lithium pretreatment of cultured ischemic retinal neurocytes decreases DNA damage by increasing NHEJ repair in *vitro*. (**a**) Retinal neurocytes were stained with Map-2 (green) and *γ*-H2AX (red) at different time points. The relative quantification of *γ*-H2AX expression following serum starvation was determined by counting foci in 50 randomly selected, Map-2-positive cells. Scale bars: 20 *μ*m. (**b**) lithium exposure caused a marked decrease in *γ*-H2AX staining (0 day, 3.03±0.27% 1 day, 23.64±1.11% 2 days, 26.47±1.67% 3 days, 31.78±2.15%) compared with the control under a serum-free condition from the first day (0 day, 3.00±0.19% 1 day, 45.51±4.44% 2 days, 46.50±4.76% 3 days, 50.99±5.17% *P<0.05). Data are graphically represented with normal and serum-free condition. *n*=3 for each group, *P<0.05. (**c**) Schematic of the NHEJ repair assay. (**d**) Rejoining capacity of retinal neurocytes with or without lithium. The plasmid substrate pEGFP-N1 was linearized with the *Eco*RI endonuclease, which cleaves between the promoter and the GFP gene and prevents GFP expression. Recircularization of the linearized plasmid was detected as GFP-positive signal via flow cytometric analysis. *n*=3 for each group, *P<0.05. (**e**) The MTT assay showed that lithium treatment also increased viability on the third day (3d) (3 days: control, 0.238±0.054; lithium, 0.269±0.015). However, in neurocytes deprived of serum, lithium-treated improved cell survival from the first day (1 day, 0.251±0.032; 2 days, 0.225±0.031; 3 days, 0.202±0.026) compared with the serum-starved controls that were not treated with lithium (1 day, 0.228±0.049; 2 days, 0.192±0.025; 3 days, 0.175±0.041). *n*=3 for each group, *P<0.05. All error bars represent SEM

To elucidate the DNA repair mechanism through which lithium provides neural protection, DNA DSBs and NHEJ efficiency was assayed *in vitro*. Primary retinal neurocytes were cultured and treated with 1.0 mM lithium chloride, and after 24 h were deprived of serum. *γ*-H2AX is a well-characterized marker of DNA DSBs *in situ* and an early marker of neuronal endangerment following seizures;^25, 26^ thus, the DNA breaks in serum-deprived neurons were detected by double staining with *γ*-H2AX and Map-2. As shown in Figure 1a, *γ*-H2AX foci were observed in Map-2-positive cells, and while the number of *γ*-H2AX foci in retinal neurocyte cells cultured in the presence of serum and treated with lithium or vehicle is no different except on the third day, in serum-deprived neurocytes, lithium exposure caused a marked decrease in *γ*-H2AX staining compared with the control under a serum-free condition from the first day (*P<0.05) (Figure 1b).

To assess NHEJ activity, as described in a previous study,^24^ primary retinal neurocytes were transfected with a NHEJ substrate to assay overall NHEJ (see Figure 1c for the structure of the NHEJ substrate and the strategy for measuring NHEJ). In this assay, a GFP reporter plasmid is linearized with a restriction enzyme and recircularization via NHEJ repair is detectable by GFP expression. In serum-starved retinal neurocytes, lithium promoted significantly more DNA NHEJ repair (2.64-fold greater) compared with control cells (*P<0.05). In contrast, lithium had no effect without serum-free stimulation (Figure 1d).

Consistent with the above results, for neurocytes grown in the presence of serum, lithium treatment also increased viability on the third day. However, in neurocytes deprived of serum, lithium treatment improved cell survival from the first day compared with the serum-starved controls that were not treated with lithium (*P<0.05; Figure 1e). Thus, lithium could promote DNA stability and survival of neurocytes when cultured in serum-free medium.

### Serum-free stimulation triggers upregulation of DNA ligase IV in lithium-medicated retinal neurocytes

**Figure 2:**
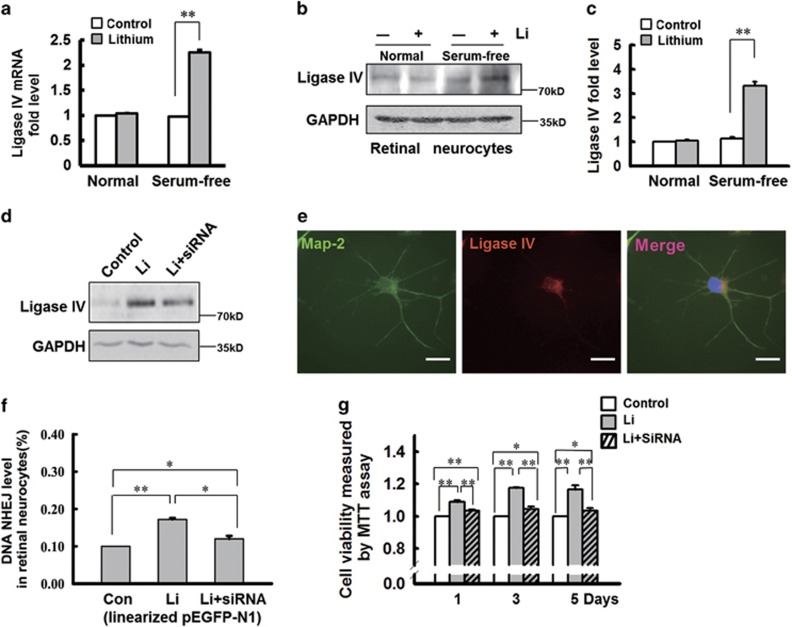
Ischemic stimulation upregulates DNA ligase IV expression in lithium-medicated retinal neurocytes. (**a**) The level of ligase IV mRNA was significantly upregulated in rat retinal neurocytes cultured in serum deprivation medium after treatment by lithium chloride. *n*=3 for each group, **P<0.001. (**b**) Western blot analysis indicates that lithium treatment upregulates ligase IV expression in rat retinal neurocytes cultured in serum-free medium but dose not affect its expression in full medium. (**c**) The relative expression of ligase IV in retinal neurocytes was quantified by densitometry. Nutritional starvation notably induced the expression of ligase IV in retinal neurocytes. In lithium-treated retinal neurocytes cultured in serum-free medium, ligase IV expression is significantly elevated. *n*=3 for each group, **P<0.001. (**d**) siRNA oligos were transfected in retinal neurocytes treated with lithium or vehicle, following serum starvation. siRNA silencing markedly decreased ligase IV expression. (**e**) Double-stained Map-2 (green) and ligase IV (red) showed that ligase IV was expressed in retinal neurocytes. Scale bars: 20 *μ*m. (**f**) Silencing of ligase IV significantly abolished the elevation of DNA NHEJ repair in retinal neurocytes following serum starvation (Con, 0.10% Li, 0.172±0.04% Li+siRNA, 0.120±0.08%). *n*=3 for each group, *P<0.05, **P<0.001. (**g**) Silencing of ligase IV also significantly attenuated the lithium-induced increase in retinal neurocyte viability following serum starvation (1 day, Con, 1; Li, 1.09±0.01; Li+siRNA, 1.035±0.005; 3 days, Con, 1; Li, 1.175±0.005; Li+siRNA, 1.045±0.015; 5 days; Con, 1; Li, 1.165±0.025; Li+siRNA, 1.035±0.015). All results were confirmed in at least three independent experiments. *n*=3 for each group, *P<0.05, **P<0.001. All error bars represent SEM

Many proteins are involved in DNA NHEJ repair (e.g., ligase IV, Mre11, Ku80), and thus their expression level was tracked in primary retinal neurocytes cultured in full or serum-free medium. Under either condition, the expression of Mre11 and Ku80 was unchanged (data not shown); however, in serum-deprived retinal neurocytes, lithium notably upregulated the mRNA (by 2.3-fold; **P<0.001; Figure 2a) and protein expression of ligase IV (by threefold; **P<0.001; Figures 2b and c). Therefore, serum-free stimulation has been shown to trigger the expression of ligase IV, which is confirmed by double staining (MAP-2 and ligase IV) (Figure 2e).

To confirm that ligase IV is involved in lithium-mediated neural protection during serum deprivation, the ligase IV expression was silenced via small interfering RNAs.^27^ As shown in Figure 2d, this markedly decreased ligase IV expression. In addition, the NHEJ assay showed that lithium promoted DNA NHEJ repair under serum-deprived conditions (the ratio of GFP-positive cells is represented as a histogram). In serum-starved cells treated with lithium, silencing of ligase IV significantly lessened the number of GFP-positive cells (**P<0.001, *P<0.05; Figure 2f). Given the drop in NEHJ repair, it was not surprising that the protective effect of lithium in serum-starved cells was reversed by the siRNA (*P<0.05, **P<0.001; Figure 2g). In addition, the TUNEL assay also indicated that lithium protects serum-starved cells from apoptosis (Supplementary Figure S6C). These results strongly support the assertion that, under serum-deprived conditions, lithium delivers neuroprotection by upregulating DNA ligase IV.

### The serum-deprived upregulation of Nrf-1 and lithium-induced increase of P-CREB1 cooperate to promote transcription of ligase IV in retinal neurocytes

The transcriptional regulation of ligase IV has never been studied in mammalian cells, thus we analyzed a series of luciferase reporter constructs driven by various lengths of the ligase IV proximal promoter region (from −1981 to +164). The region between −121 and −301 contains a putative binding site for activating transcription factor (ATF). (Supplementary Figure S1).

**Figure 3:**
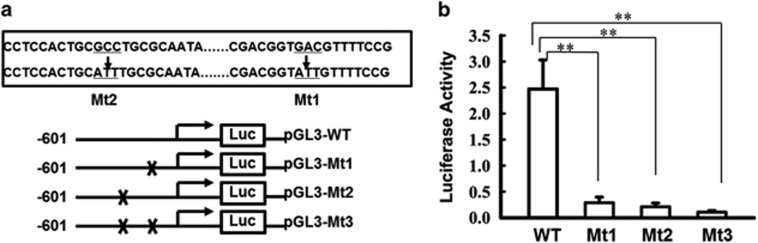
Two sites (−126, −172) are necessary for ligase IV promoter activation in retinal neural cells. (**a**) Two mutations were introduced into the ATF sites in the −601/+164 constructs. (**b**) Primary retinal neurocytes were cultured in serum-free medium, with 1.0 mM lithium, and transfected with a ligase IV promoter reporter containing the WT or Mut ATF sites, shown in (**a**). The levels of luciferase activity were normalized to *Renilla* luciferase (WT, 2.47±0.56; Mt1, 0.286±0.11; Mt2, 0.208±0.072; Mt3, 0.106±0.029). *n*=3 for each group, **P<0.001. All error bars represent SEM

The region of the ATF promoter between −301 and −121 was scanned *in silico* for transcription factor binding motifs. Two putative ATF sites were detected, at positions −126 to −133 and −172 to −183. The 5'-GTGACGTT-3' motif, at −126, is a P-CREB1 DNA-binding sequence.^28^ The other, at −172 5'-TGCGCCTGCGCA-3', is a well-known nuclear respiratory factor 1 (Nrf-1) DNA-binding sequence.^29^ To determine the role of the putative ATF sites in ligase IV promoter activation, these sites were mutated (5′-GTGCCGTT-3′ to 5′-GTATTGTT-3′, 5′-TGCGACTGCGCA-3′ to 5′-TGCATTTGCGC-3′ Figure 3a) and the entire promoter region from −601 to +164 was assayed for lithium inducibility in primary retinal neurocytes cultured in serum-free medium, with 1.0 mM lithium.

Three versions of the promoter were compared with the WT sequence: Mt1, which was mutated at the P-CREB binding site (−126/−133); mutant 2 (Mt2), which was mutated at the Nrf-1 binding site (−172/−183); and Mt3, which was mutated at both (Figure 3a). The promoter activity was compared relative to that of the backbone PGL3-Basic plasmid, showing that the Mt1 and Mt2 mutations significantly reduced the luciferase activity relative to the WT promoter sequence, and mutating both sites reduced transcription by ~90% (**P<0.001). Although the inducibility of Mt1, Mt2 and Mt3 differed slightly, the difference was not statistically significant (Figure 3b). Therefore, these data show that the sites are necessary, but not synergistic, in regulating ligase IV, and that under serum-deprived conditions they confer lithium inducibility.

**Figure 4:**
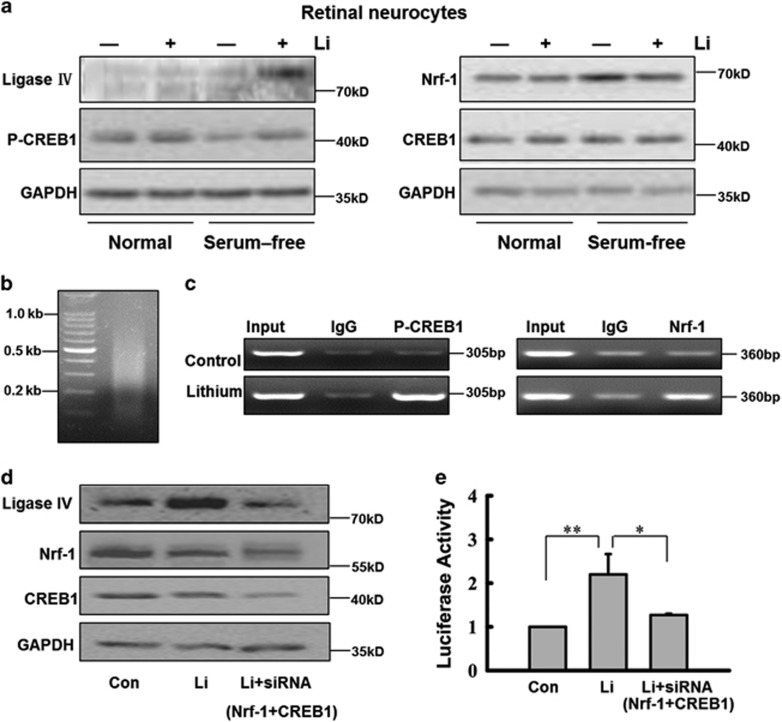
Western blot, CHIP assay and siRNA silencing demonstrated that Nrf-1 and P-CREB1 cooperate to promote transcription of ligase IV in retinal neurocytes. (**a**) Retinal expression pattern of Nrf-1, P-CREB1 and ligase IV in retinal neurocytes *in vitro*. Western blotting indicates that lithium (1.0 mM) alters the expression level of Nrf-1, P-CREB1 and ligase IV in retinal neurocytes only under conditions of serum starvation. (**b**) Sonicated DNA isolated from retinal neurocytes treated with formaldehyde to crosslink endogenous proteins to DNA (fragments averaged ~0.5 kb in length). (**c**) CHIP assayed for P-CREB1, Nrf-1 antibody and a control normal rabbit IgG, and PCR amplified a 360- or 305-bp fragment of ligase IV promoter spanning the ATF sites. The amplified PCR fragments were analyzed on a 2% agarose gel. (**d**) siRNA silencing markedly decreased CREB1 and Nrf-1 expression in retinal neurocytes. In accordance, the siRNA abolished the lithium-stimulated increase in ligase IV expression. (**e**) Silencing of CREB1 and Nrf-1 significantly inhibited the increase in promoter activity in lithium-treated cells (Con, 1; Li, 2.2±0.47; Li+siRNA, 1.27±0.03). *n*=3 for each group, *P<0.05, **P<0.001

We measured the expression level of Nrf-1, CREB1, P-CREB1 and ligase IV in primary retinal neurocytes treated with lithium or vehicle by western blotting. In full medium, CREB1 and P-CREB1 were highly expressed in primary retinal neurocytes, and treatment with lithium did not alter the expression of CREB, P-CREB1, Nrf-1 and ligase IV (Figure 4a). Serum deprivation, however, caused a significant decrease in CREB1 phosphorylation but not CREB1, and remarkably induced the expression of Nrf-1, but ligase IV expression was negligible. Pretreating the cells with lithium, however, notably boosted CREB1 phosphorylation under a serum-free condition. These results are consistent with the previous reports that showed serum deprivation in neural cells induces upregulation of Nrf-1^30^ and downregulation of P-CREB1.^31^ Long-term treatment with lithium alone does not significantly increase either P-CREB1 levels or CRE-driven gene transcription, this effect is only seen with glutamate or ischemic insult.^32^ In retinal neurocytes treated with lithium, robust CREB1 phosphorylation and Nrf-1 highly upregulated ligase IV compared with the control under a serum-free condition. In addition, compared with control cells, lithium-treated cells expressed less Nrf-1 under a serum-free condition. We posit that the decrease in Nrf-1 was the result of lithium-mediated neuroprotection.

CHIP analysis then was used to further examine whether Nrf-1 and P-CREB1 are directly involved in lithium-mediated transcriptional control. At 24 h after treatment with 1.0 mM lithium, retinal neurocytes were cultured in serum-free medium. At 48 h after treatment, chromatin was sonicated into fragments of ~0.5 kb in length (Figure 4b) and precipitated with rabbit antibodies to Nrf-1 and P-CREB1 (or normal rabbit IgG). The precipitated DNA was subjected to PCR amplification using primers designed to amplify a 360- and 305-bp fragment of the ligase IV promoter region flanking the ATF site. As shown in Figure 4c, the 360- and 305-bp DNA fragments were amplified from the precipitates of retinal neurocytes treated with lithium, whereas in retinal neurocytes treated with vehicle, DNA was only weakly detected. These results indicate that P-CREB1 and Nrf-1 bind to the promoter region of ligase IV. Thus, our data suggest that Nrf-1 and P-CREB1 nonsynergistically upregulate the expression of ligase IV. Moreover, the efficiency of binding of P-CREB1 and Nrf-1 is increased in retinal neurocytes exposed to lithium (Figure 4c).

Furthermore, we performed RNA interference with effective siRNA oligos according to the previous study.^33, 34^ Silencing CREB1 and Nrf-1 notably abolished the lithium-stimulated increase of ligase IV expression (Figure 4d). The inducibility of the wild-type p(−601/+164) ligase IV luciferase reporter constructs showed that silencing CREB1 and Nrf-1 could significantly inhibit the increase in promoter activity observed in the wild-type oligos following lithium treatment (*P<0.05, **P<0.001; Figure 4e). Moreover, the luciferase assay also indicated that the promoter activity was enhanced by PKA activators (forskolin) and was inhibited by PKA inhibitors (H89) (Supplementary Figure S2F). Thus, two sites (−126, −172) are necessary for ligase IV promoter activation in retinal neural cells.

Thus, the results above suggest that the serum-deprived upregulation of Nrf-1 and lithium-induced increase in P-CREB1 cooperate to promote transcription of ligase IV in retinal neurocytes. Transcriptional regulation of ligase IV by P-CREB1 is also confirmed by PKA activators (forskolin) and PKA inhibitors (H89) (Supplementary Figures S2C–E). In addition, P-CREB1 is highly expressed in neurocytes cultured in full medium. Therefore, the serum-deprived upregulation of Nrf-1 might trigger transcription of ligase IV, which is confirmed by exogenous Nrf-1 (Supplementary Figures S2A and B).

### Retinal expression pattern of Nrf-1, P-CREB1 and ligase IV *in vivo* suggests that ischemic stimulation triggers lithium-mediated upregulation of ligase IV

**Figure 5:**
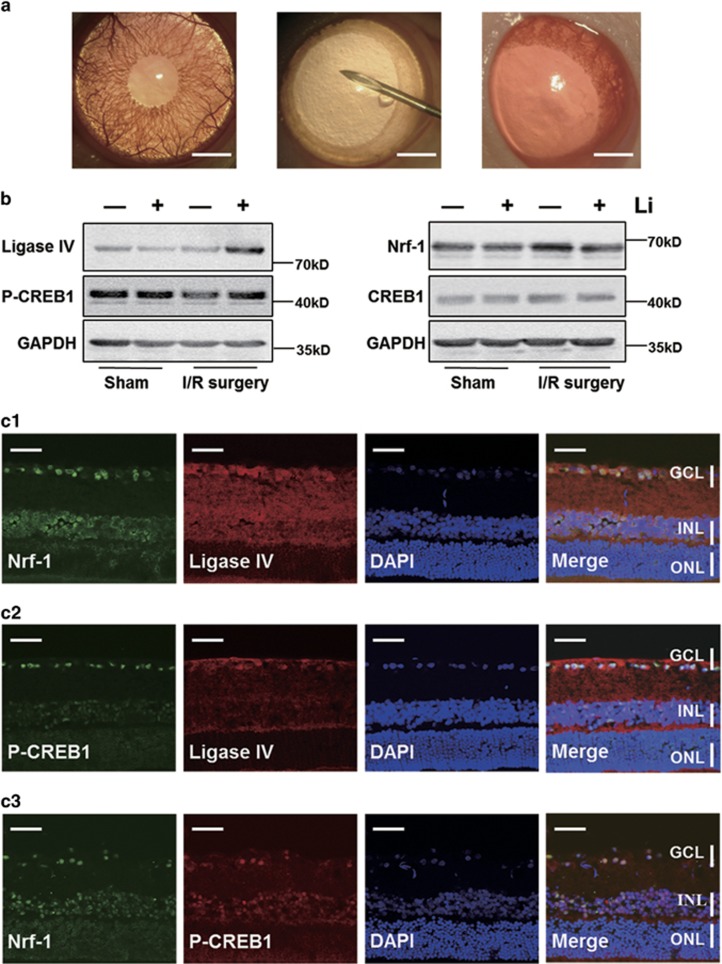
Ligase IV, P-CREB1, CREB1 and Nrf-1 expression pattern in rat retina after I/R surgery. (**A**) The image of normal rat eye, rat eye at I/R surgery and the rat eye after I/R surgery. The normal rat eyes have the characteristic of transparency cornea, normal bloodstream in iris and fundus red light reflection (left); the eye at I/R surgery is of corneal edema, interruption of blood flow in fundus and pale retina (middle); the bloodstream of iris and fundus recovery immediately right after the I/R surgery (right). Scale bars: 1000 *μ*m. (**B**) Western blot shows lithium only alters the expression levels of Nrf-1, P-CREB1 and ligase IV, in retinal tissue subjected to I/R surgery. (**C**) Location of expression of P-CREB1, Nrf-1 and ligase IV in retina after following I/R surgery. Double staining indicates that ligase IV, Nrf-1 and P-CREB1 are located in same position of retina, mainly expressed in INL and GCL. At 24 h after I/R surgery, sections of lithium-treated retina were double stained with antibodies recognizing Nrf-1 (green) and ligase IV (red) (c1), P-CREB1(green) and ligase IV (red) (c2), and Nrf-1 (green) and P-CREB1 (red) (c3), respectively. Scale bars: 50 *μ*m

To test whether the expression changes observed in cultured cells faithfully reflects the scenario in the live retina, the expression of Nrf-1, CREB1, P-CREB1 and ligase IV *in vivo* was tracked in rat I/R surgery model (Figure 5A). Over the course of I/R surgery, the P-CREB1, Nrf-1 and ligase IV expression patterns reflected our *in vitro* results (Figure 4a). In normal, healthy adult retina, the expression of ligase IV and Nrf-1 was negligible, whereas P-CREB1 was highly expressed (Figure 5B and Supplementary Figure S5). Lithium treatment had no effect without I/R stimulation. Similarly, I/R surgery markedly elevated Nrf-1 expression and decreased CREB1 phosphorylation. Notably, in eyes subjected to I/R surgery, lithium pretreatment induced upregulation of P-CREB1 and ligase IV, and decreased Nrf-1.

The expression patterns were tested by immunohistoflourescence, confirming that after I/R surgery, double staining indicated ligase IV, Nrf-1 and P-CREB1 expression colocalized, and was strongest in the inner layer (INL) and ganglion cell layer (GCL) (Figure 5C). Thus, the results *in vivo* are consistent with that *in vitro*.

### Lithium enhances DNA NHEJ repair and light responses *in vivo*

**Figure 6:**
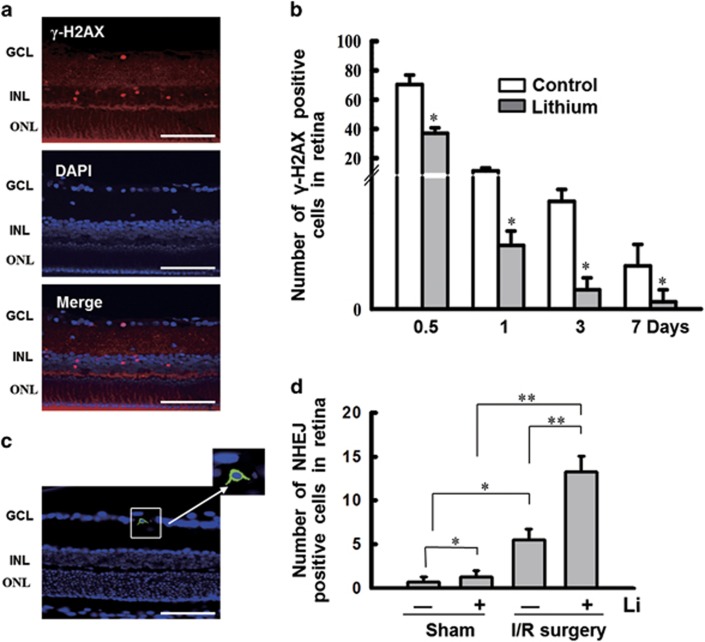
Lithium treatment stimulates NHEJ repair in a rat model of retinal I/R injury. (**a**) Location of *γ*-H2AX foci coincides with CREB1 phosphorylation, Nrf-1 and ligase IV in retina after following I/R surgery. The *γ*-H2AX-positive cells were primarily located in the INL and GCL. Scale bars: 100 *μ*m. (**b**) *γ*-H2AX expression was quantified after I/R surgery by counting foci. These data are graphically represented (12 h, 37.0±3.74; 1d, 2.66±0.6; 3d, 0.8±0.5; 7d, 0.3±0.5) compared with controls (12 h, 70.33±6.55; 1d, 11.3±0.2; 3d, 4.5±0.5; 7d, 1.8±0.9). (**c**) DNA NHEJ assay *in vivo*. (The strategy of the NHEJ repair assay is shown in Figure 3d.) At 24 h after I/R surgery, intravitreal injection was performed with the linearized plasmid pEGFP-N1 following transfecting reagent following the manufacturer's instructions (*in vivo*-jetPEI). At 48 h after transfection, the retina is fixed and sliced. GFP is detectable in the RGC layer. Scale bars: 100 *μ*m. (**d**) A comparison of GFP-positive cells in the rat retina. Lithium pretreatment promotes DNA NHEJ activity (Sham, 0.67±0.62; Sham+Li, 1.25±0.72; I/R surgery, 5.5±1.19; I/R surgery+Li, 13.25±1.83). *n*=8 for each group, *P<0.05. **P<0.001. All error bars represent SEM

We then tracked *γ*-H2AX foci in a time course in retina subjected to I/R surgery. Here, *γ*-H2AX foci were more abundant, and *γ*-H2AX-positive cells were primarily located in retinal GCL and INL (Figure 6a). Moreover, in retina pretreated with lithium, *γ*-H2AX foci were far less abundant (*P<0.05; Figure 6b). The presence of *γ*-H2AX foci in retinal GCL and INL agreed with the expression patterns of P-CREB1, Nrf-1 and ligase IV. In addition, TUNEL assay also indicated that lithium significantly decreased the number of apoptosis cells in retina at different time points after I/R surgery (Supplementary Figures S6A and B). Moreover, the rat retina HE staining also showed that lithium protected the thickness of rat retina from getting thinner after I/R surgery (data not shown).

Moreover, the effect of lithium on DNA NHEJ activity was directly tested in the ischemic rat retina. After I/R surgery, the NEHJ substrate, a linearizing pEGFP-N1 was delivered, as described in Materials and Methods. At 48 h after transfection, eyeballs were fixed, sliced and examined for GFP expression. As expected, the RGC layer showed the most activity (Figure 6c), and, with I/R surgery, lithium significantly promoted DNA NHEJ activity (*P<0.05; Figure 6d). Interestingly, lithium also promoted NHEJ even without I/R surgery, improving efficiency by 1.83-fold (*P<0.05). We speculate that this effect is observed because transfection causes mild damage to cells in RGC layer. In addition, in the I/R surgery group, lithium promoted DNA NHEJ activity by 2.4-fold (**P<0.001). I/R surgery also markedly enhanced NHEJ events even without lithium pretreatment (**P<0.001). These results are partly consistent with previous reports showing ischemic stimulation spontaneously activates DNA repair,^3, 6^ and that lithium promotes DNA repair under the pathological, ischemic state.^35, 36^

**Figure 7:**
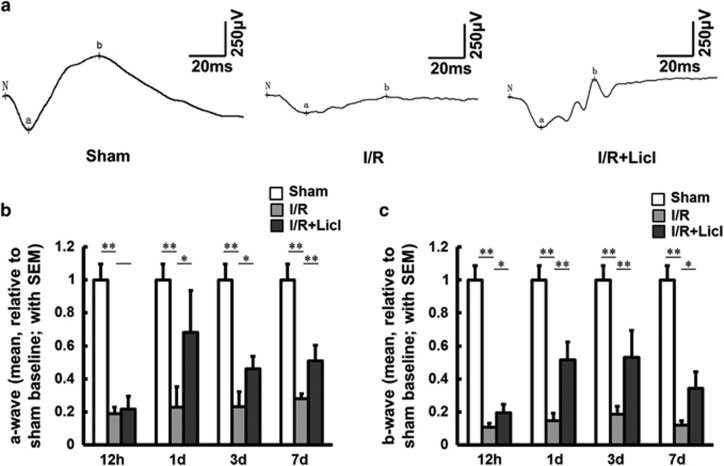
ERG tests show lithium pretreatment improves electrophysiological function in a rat model of I/R surgery. (**a**) Representative traces from sham-(left panel), vehicle-(middle panel) and lithium (right panel)-treated rats 3 days after I/R surgery. (**b**) Relative quantification of a-wave to sham baseline at various time points after I/R surgery (2.5 cd•s/m^2^; sham eyes, 1±0.095; vehicle-treated eyes, 1d, 0.227±0.124; 3d, 0.232±0.09; 7d, 0.28±0.03; lithium-treated eyes, 1d, 0.681±0.254; 3d, 0.461±0.076; 7d, 0.51±0.092). (**c**) Relative quantification of b-wave to sham baseline at the same time points (sham eyes, 1±0.086; vehicle-treated eyes, 12 h, 0.108±0.024; 1d, 0.146±0.044; 3d, 0.184±0.049; 7d, 0.118±0.028; lithium-treated eyes, 12 h, 0.195±0.05; 1d, 0.515±0.11; 3d, 0.529±0.164; 7d, 0.343±0.1). *n*=8 for each group, *P<0.05. **P<0.01. All error bars represent SEM

Finally, scotopic electroretinogram (ERG) was used to examine retinal function. The amplitudes and implicit times of ERG a- and b-waves were examined at different time points after I/R surgery. In comparison with sham eyes, a- and b-wave amplitudes were markedly decreased after I/R surgery. A representative intensity-response series recorded in dark-adapted, sham-, vehicle- and lithium-treated rats is shown in Figure 7. At 12 h after I/R injury, the a-wave amplitudes of vehicle- and lithium-treated rats were similar; in lithium-treated eyes however, from days 1 to 7, the amplitudes increased significantly (*P<0.05, **P<0.01) (Figure 7b). Moreover, after surgery, lithium pretreatment also significantly increased the b-wave, for all time points (*P<0.05, **P<0.01) (Figure 7c). Taken together, these results firmly support the idea that DNA breaks occur in the ischemic retina, and pretreatment with lithium promotes NHEJ repair of these DNA breaks. Lithium pretreatment attenuates damage to the retina after I/R surgery.

## Discussion

Our results in retinal neurocytes uncover a previously unidentified property of lithium, showing it promotes DNA stability and survival of ischemic retinal neurocytes by upregulating DNA ligase IV. Moreover, ligase IV is transcriptionally regulated by the ischemic upregulation of Nrf-1 and lithium-induced increase P-CREB1 in retinal neurocytes. Importantly, ischemic stimulation triggers the upregulation of ligase IV. All results have been confirmed in rat retina following I/R injury. Thus, our findings provide direct biochemical and functional evidence to support the idea that ischemia-induced damage might activate DNA NHEJ repair and, in the retina, lithium pretreatment boosts this process and so protects the retina.

Many previous studies have investigated lithium-mediated neuroprotection and its mechanism. Our data show that *γ*-H2AX foci was observed in retinal cells both *in vitro* and *in vivo* with ischemic stimulation. The DNA rejoining levels both *in vitro* and *in vivo* reveal that, after ischemia, lithium-pre-treated retinal neurocytes showed ~2-fold more DNA repair (Figures 1d and 6d). Notably, however, lithium did not improve cell viability as much as it promoted DSB repair (Figure 1e). Thus, ischemic stimulation likely damages the cell in ways other than just by inducing DNA DSBs. Still, promoting DNA DSB repair might be one pathway that lithium supports in giving neuroprotection under ischemic condition.

Yang *et al.*^24^ reported that lithium-mediated protection of hippocampal cells involves enhancement of DNA-PK–dependent repair in mice but did not identify a particular protein target for lithium. Our study in retinal neurocytes showed that lithium did not directly affect the expression of DNA-PK (data not shown), but rather influenced the expression of ligase IV, a key protein in the predominant pathway for repair of DNA DSBs in postmitotic cells. More importantly, lithium treatment affected the expression level of ligase IV in retinal neurocytes both *in vitro* and *in vivo*, under nutritional starvation or an ischemic condition (Figures 2a–c,4a and 5b). In addition, our data indicated that human retinoblastoma cells did not respond to lithium-mediated retinal neuroprotection by ligase IV. Ligase IV expression in starved WERI-Rb1 cells also did not change with lithium treatment (Supplementary Figure S3). Therefore, the effect of lithium in enhancing ligase IV was specific to primary neural cells under nutritional starvation or an ischemic condition.

In mice, ligase IV deficiency causes embryonic lethality.^37^ Ligase IV is markedly downregulated in the retina of the P5 rat and almost silenced in the adult retina (Supplementary Figure S4). Previous reports on cerebral development demonstrated the DNA repair activity in the young and adult rat, but the expression level declined with age. Nuclear extracts from aged CNS tissue contained NHEJ activity that could be supplemented by exogenously supplied T4 ligase and DNA polymerase b.^38, 39^ In addition, ligase IV associates with DNA-PK in a DNA-independent manner.^40^ Therefore, our discovery aligns well with earlier reports.^21, 22, 24^

Our discoveries concerning the transcriptional mechanism of ligase IV support the idea that, in these retinal cells, the bioactivity of lithium in promoting DNA NHEJ repair might be triggered by ischemic insult. Our results show that Nrf-1 and P-CREB1 are essential to transcription of ligase IV. These results are partly supported by previous reports. CREB is a broadly expressed leucine-zipper-containing transcription factor affecting neuron survival, axon growth, and synaptic transmission. P-CREB may be a more appropriate predictor of CRE-driven transcriptional activity.^41, 42^ In nutritionally starved neurocytes, lithium might significantly attenuate a decrease in CREB1 phosphorylation. The other key factor, Nrf-1, is essential for embryogenesis, and embryos homozygous for Nrf-1 disruption die between embryonic days 3.5 and 6.5. Nrf-1 is likely involved in transcriptional regulation of multiple target genes, including those associated with mitochondrial proteins, DNA DSBs and cell cycle.^43^ In addition, the NRF proteins are important for upregulating antioxidant and xenobiotic-metabolizing enzymes and the regulation of neurite outgrowth during oxidative stress.^44, 45^ After 36 h of lithium treatment, primary bovine aortic endothelial cells upregulate the mRNA level of Nrf-1 by twofold.^46^

More importantly, our data suggest not only that Nrf-1 and P-CREB1 positively and necessarily promote ligase IV transcription but also that Nrf-1 triggers the process. Our results showed that, in the retina, from first day after birth to adulthood, Nrf-1 and ligase IV display the same expression pattern. The expression pattern we observed was consistent with previous reports showing that NRF is expressed ubiquitously throughout the developing retina and central nervous system in zebrafish at 24 h after birth. However, at 48 h after birth, the expression of NRF was detected in the ganglion cell layer, in the neurons of the inner nuclear layer and in the optic nerve and optic tracts, but it was no longer detectable by *in situ* hybridization by 72 h.^47^ In contrast, we found that the level of CREB1 phosphorylation was almost constant in the adult retina, and the protein could be detected in nuclei of the INL and GCL, but not in the outer nuclear layer (ONL). Furthermore, I/R surgery causes most of the damage to the INL and GCL.^48^ The CREB1 phosphorylation, Nrf-1 and ligase IV primarily in the INL and GCL mirrors that of *γ*-H2AX foci. Thus, our discoveries of ligase IV transcription also support the idea that ischemia-induced damage might activate DNA NHEJ repair mediated by lithium in retinal neuroprotection.

**Figure 8:**
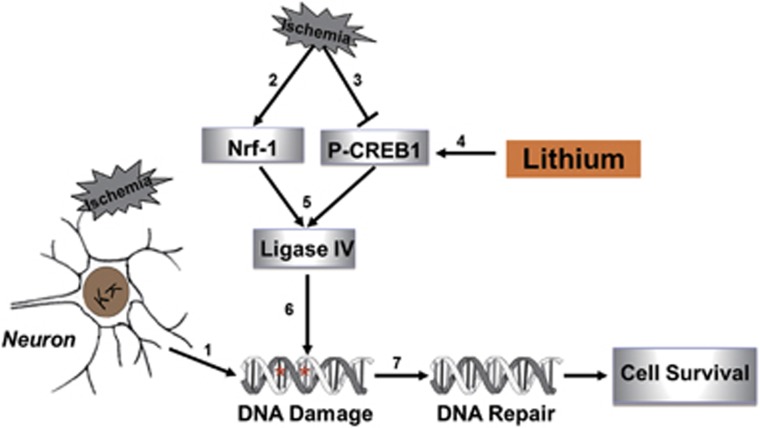
Ischemia-induced damage activates DNA NHEJ repair in the lithium-medicated retina. (1) Ischemia induces DNA damage in retinal neurocytes. (2) Ischemic damage upregulates the transcription factor Nrf-1. (3) Ischemic damage downregulates the transcription factor P-CREB1. (5) P-CREB1 and Nrf-1 positively regulate ligase IV gene expression. (6) Ligase IV binds to the break sites of genomic DNA in retinal neurocytes. (7) Ligase IV promotes DNA NHEJ repair and cell survival

In addition, the effects of lithium on P-CREB have a significant impact on CRE-driven gene expression in ischemia-treated cells following long-term lithium exposure. Moreover, Nrf-1 is known to control the expression of other genes.^9, 49, 50^ Thus, in neurons, regulation of DNA NHEJ repair might be one of several complex signaling pathways that respond to lithium. Thus, our results identify one mechanism through which the bioactivities of lithium might offer neural protection (Figure 8): If retinal neurocytes become ischemic and suffer genomic DNA instability, Nrf-1 expression is triggered; however, P-CREB1 is downregulated. Pretreatment with lithium upregulates the expression of P-CREB. Thus, although P-CREB1 and Nrf-1 both positively and necessarily regulate ligase IV gene expression, Nrf-1 triggers the process of neuroprotective DNA NHEJ repair mediated by lithium.

In summary, although it is known that lithium promotes neuroprotection from ischemia-induced neuronal damage, the drug's therapeutic mechanisms have remained obscure. The present study provides substantial evidence that, to offer neuroprotection in the face of ischemia-induced damage, lithium might promote DNA NHEJ repair by upregulating ligase IV. This study should not only provide new insights into lithium-medicated neuroprotection but also might extend the application of lithium in clinical therapy.

## Materials and Methods

### *In vitro* experiment

#### Primary rat retinal neurocytes cultures and drug treatment

Retinas were dissociated enzymatically to make a suspension of single cells, essentially as described previously.^11^ Briefly, P1-day-old SD rats were killed by an intraperitoneal injection of Nembutal (60 mg/kg) (Sigma-Aldrich, St Louis, MO, USA). The retinas that were separated from the enucleated eyeballs were incubated for 20 min at 37 °C in a solution containing 0.125% trypsin to dissociate their cells. To yield a suspension of single cells, the tissue was then triturated sequentially through a narrow-bore Pasteur pipette in a solution of Dulbecco's modified Eagle's medium (DMEM) containing 10% fetal bovine serum (FBS). The cells were then seeded at a density of ~1 × 10^6^ cells per ml on the culture plate precoated with 0.01% poly-l-Lysine for 2 h, and incubated at 37 °C in a humidified atmosphere of 5% CO_2_ and 95% air. After 12 h, the cells were treated with 10 *μ*m/ml Ara-C (Sigma) to suppress the growth of non-neurocytes. After the cells were cultured for 12 h and treated as described above, the media were replaced with complete medium (10% FBS). The cultured cells were characterized by staining with Map-2 antibody (Boster, Wuhan, China).

At 3 days after culture, the cells were incubated in the presence or absence of lithium chloride, respectively. At 24 h after incubation, the culture media were replaced with serum-free media to initiate nutrient-deprived state in ischemia in one group. For controls, the cells were cultured in full media with or without lithium chloride. The media were changed every two days.

Human retinoblastoma cells, the WERI-Rb1 cell line, rat glioma C6 cells (ATCC, Manassas, VA, USA) and the mouse retinal cell lines ( RGC-5, a generous gift of Dr Zhiqun Tan; 661W, a generous gift of Dr Xuri Li's laboratory) were cultured in DMEM (Gibco, Carlsbad, CA, USA) supplemented with 10% FBS (Gibco) and 1% penicillin/streptomycin (Gibco) in a humidified 5% CO_2_ incubator.

#### Reporter and plasmid construction

A fragment spanning from −1981 to +164 relative to the transcription start site of rat ligase IV genomic sequence (GeneBank: NC_005115) was produced by PCR with the forward primer 5′-GGGGTACCCCTTGCGCCGCGGCTGGCGCAGC-3′ and the common reverse primer 5′-GAAGATCTTCAACCTGTAAATCCCAGTCCAG-3′. This fragment was fused to the promoterless firefly luciferase gene of pGL3-Basic vector (Promega, Madison, WI, USA) to generate a ligase IV (−1981/+164)-luc. A series of reporter constructs with the same 3′ ends but with different 5′ ends were also constructed by PCR using pGL3 (−1981/+164)-luc as the template and the common reverse primer as mentioned above. The forward primers used were as follows: 5′-GGGGTACCCCAGCCTCTAGTCCATGTAAGCA-3′ for ligase IV (−601/+164)-luc; 5′-GGGGTACCCCGCGCAGGCGCACTGAGCCGCC-3′ for ligase IV (−301/+164)-luc; 5′-GGGGTACCCCGTCGGAATGAAAGTGGGCGAC-3′ for ligase IV (−121/+164)-luc; and 5′-GGGGTACCCCCCCTCTGCCGCCTAGCTACCT-3′ for ligase IV (+91/+164)-luc.

The first mutant (Mt1, GTGACGTT to GTATTGTT) was introduced for ligase IV (−601/+164)-luc by digestion and adding an inserter. The construct of ligase IV (−601/+164)-luc was digested by *Pvu*II and *Avr*II, and then ligated with the inserter containing the mutation, 5′-CTGGCGGAAACGCGGGTTTGGCCTCGACGGTATTGTTTTCCGGTCGGAATGAAAGTGGGCGACTTCTCGGGAGGCTGC-3′. The subclone was tested by the specific primers (forward, 5′-GGTTTGGCCTCCGACGGTATTG-3′ reverse, 5′-AACCTGTAAATCCCAGTCCAG-3′). The second mutation (Mt2, 5′-TGCGCCTGCGCA-3′ to 5′-TGCATTTGCGC-3′) of ligase IV promoter fragments was introduced to the ligase IV (−601/+164)-luc plasmid. The point mutation construct was created using a Site Directed Mutagenesis Kit (Beyotime, Jiangsu, China). The primers are designed using our web-based QuikChange Primer Design Program available online at www.agilent.com/genomics/qcpd. The forward primer sequences were as follows: 5′-AGGCTCTCTGGCCTCCACTGCATTTGCGCAATAAACAGC-3′, 5′-GCTGTTTATTGCGCAAATGCAGTGGAGGCCAGAGAGCCT-3′. The method in detail was followed the protocol of the kit. The mutated nucleotide sequences of all mutant constructs were confirmed by DNA sequencing. The third mutation (construct Mt3, 5′-GTGACGTT-3′ to 5′-GTATTGTT-3′ and 5′-TGCGCCTGCGCA-3′ to 5′-TGCATTTGCGC-3′) is contained Mutant 1 and mutant 2 in ligase IV promoter fragments The protocol is followed the mutant 2 (Mt2). However, the plasmid ligase IV (−601/+164)-luc with mutant 1(Mt1) is used as PCR template (Mt1,the bases GAC were changed to ATT to make the site of P-CREB1 inactive and did not affect the function of the other sequence; Mt2,the bases GCC were changed to ATT to make the site of Nrf-1 inactive and did not affect the function of the other sequence; Mt3, both the sites of P-CREB1 and Nrf-1 were inactive). All of the constructs were confirmed by DNA sequencing.

The plasmid pEPI-Nrf-1 was derived from pEPI-GFP (generously provided by Dr HJ Lipps), which rat Nrf-1 cDNA was inserted in pEPI-GFP by restriction digested sites, *Eco*RI and *Bam*HI.

#### Ligase IV promoter-reporter assay

Rat glioma C6 cells and primary rat retinal neurocytes were transfected using Lipfectin2000 (Invitrogen, Carlsbad, CA, USA) as described previously.^11, 12^ Plasmids transfected contained 2 *μ*g of various reporter plasmids, 2 *μ*g of expression plasmids or pcDNA3-based vectors and 100 ng of *Renilla* luciferase reporter plasmid, pCMV-RL (Promega, Madison, WI, USA). The pCMV-RL plasmid encoding *Renilla* luciferase was included in all the samples to monitor the transfection efficiency. At 24 h post-transfection, the levels of firefly and *Renilla* luciferase activity were measured sequentially from a single sample using the Dual-Glo Luciferase Assay System (Promega). The levels of firefly luciferase activity were normalized to *Renilla* luciferase activity.

#### MTT assay

Cell viability was determined by MTT assay at different time points after ischemic insult and lithium chloride treatment. Cell viability was the optical density ratio of a treated culture over an untreated control.

#### Western blotting

Western blotting assay was carried out as a standard protocol. The following antibodies were used: CREB1 (Cell Signaling Technology, Beverly, MA, USA), P-CREB1 (Cell Signaling Technology), Nrf-1 (Santa Cruz, CA, USA) and ligase IV (Santa Cruz), respectively. The membrane was incubated with horseradish peroxidase-conjugated secondary anti-rabbit antibody (Cell Signaling Technology). GAPDH (Proteintech, Chicago, USA) served as a loading control. Protein bands were detected using an Enhanced Chemiluminescence Detection System (Millipore, Billerica, MA, USA).

#### Immunofluorescence analysis of cells

Immunofluorescence assay was carried out as standard protocol. For the assay of DNA damage, cells were fixed and analyzed for *γ*-H2AX foci formation by immunofluorescence with anti-phospho-H2AX ser-139 antibody (Millipore) and anti-Map-2 (Boster). Images were captured using a fluorescence microscope (Carl Zeiss, MicroImaging GmbH, Göttingen, Germany). The amount of *γ*-H2AX foci was scored in images obtained using a constant exposure time. At least 600 cells were counted.

#### CHIP assay

CHIP was performed using the CHIP Assay Kit (Upstate Cell Signaling Solutions, Lake placid, NY, USA) according to the manufacturer's instructions. The following antibodies were used: Nrf-1 (Santa Cruz) and P-CREB1 (Cell Signaling Technology), respectively, or normal IgG (Sigma-Aldrich). PCR amplification were performed using the primers spanning the ATF site on the ligase IV promoter (P-CREB1, forward, 5′-GTCGGAATGAAAGTGGGCGAC-3′ reverse, 5′-TATCAGAACAAGATTTCCTC-3′ Nrf-1, forward, 5′-CCAGGCTCTCTGGCCTCCAC-3′ reverse, 5′-TATCAGAACAAGATTTCCTC-3′).

#### RNA interference

The siRNA sequences used for targeted silencing of ligase IV, Nrf-1, CREB1 and control sequences were as follows – ligase IV: 5′-TTGCTCAATTTACCAAGAGDTDT-3′^25^ CREB1:^34^ 5′-GCACTTAAGGACCTTTACTDTDT-3′; Nrf-1 (the pool), Nrf-1-siRNA-1:^33^ 5′-GAAAGCTGCAAGCCTATCTDTDT-3′ Nrf-1-siRNA-2: 5′-GCCACAGGAGGTTAATTCADTDT-3′; Nrf-1-siRNA-3: 5′-GCATTACGGACCATAGTTADTDT-3′; Nrf-1-siRNA-4:

5′-AGAGCATGATCCTGGAAGADTDT-3′ and control, 5′-GGUUUGGCUGGGGUGUUAUDTDT-3′. The oligos were purchased from Ribo**b**io (Guangzhou, China). The retinal neurocytes (4 × 10^6^ cells per well in 6-well culture plates) in good condition underwent lipid-mediated transfection with Lipofectamine RNAiMAX (Invitrogen), as recommended by the manufacturer. Transfections were performed two times, 24 h apart. Expression levels of Nrf-1, CREB1 and ligase IV were measured by western blot at 48 h after transfection.

#### Real-time RT-PCR

Total RNA was isolated with TRIzol Reagent (Invitrogen). One microgram of total RNA was subjected to reverse transcription with the Iscript cDNA Synthesis Kit (Bio-RAD, Hercules, CA, USA) following the manufacturer's protocol. Expression levels of Nrf-1 and ligase IV were measured by real-time RT-PCR by using the Roche 480 System (Roche, Indianapolis, IN, USA). The following primer pairs were used: for Nrf-1, 5′-TAGCCCATCTCGTACCATCAC-3′ and 5′-TTTGTTCCACCTCTCCATCAG-3′ for ligase IV, 5′-GGCACTTCAAGGAGTTTCTGGA-3′ and 5′-ATGTAAAGCTTAGCCAGCATGG-3′ for *β*-actin, 5′-TCACCCACACTGTGCCCAT-3′ and 5′-TCTTTAATGTCACGCACGATT-3′.

### NHEJ assay *in vitro*

Primary rat retinal neurocytes were suspended and transfected with the linearized plasmid pEGFP-N1 digested with *Eco*R1 using the AMAXA Electroporation System (Lonza, Levallois-Perret, France). The intact pEGFP-N1 was used as the positive control and treatment with pcDNA3.1 was used as the negative control. After 48 h, the cells were harvested and the staining fluorescence intensity was measured using a BD FACSort flow cytometer (BD Biosciences, San Jose, CA, USA). The green fluorescent cells represented the NHEJ of the repaired DSBs and the restoration of GFP expression. For each analysis, 200 000 cells were processed.

### *In vivo* experiment

#### Animals

A total of 40 adult Sprague–Dawley rats weighing 250–300 g were obtained from the Laboratory Animal Center, Southern Medical University (Guangzhou, China). All experimental procedures were approved through the Ethical Committee of Sun Yat-Sen University (2014–008). All animals were maintained on a light schedule of alternating 12-h periods of light and dark with free access to food and water for at least 3 days before the experiment and during the experimental period.

Time of treatment and dose of lithium were chosen based on published data^15, 20, 24^ and our pilot experiments. A range of concentration of lithium chloride (0.5–3 mEq/kg) was tested in preliminary experiments to find the most effective dose (data not shown). Experiments were performed in two groups (20 rats for each group): the experimental group and the control group. The experimental group received subcutaneous injection of lithium chloride (Sigma-Aldrich) once a day, starting from 1 week before ischemia and ending on the day of tissue sampling, whereas the control group received the same amount of PBS only. Daily dose of lithium chloride was 1.0 mEq/kg. An I/R experiment was carried on the right eyes, with the left eye as self-control. The rats were killed for tissue sampling on the 12 h, 1st, 7th and 14th day after ischemia insult, respectively.

#### I/R experiment

Rats were anesthetized with an intraperitoneal injection of a mixture of 50 mg/kg ketamine and 25 mg/kg chloropromazine. After applications of topical alcaine (0.5% proparacaine) and tropicamide (0.5%), the anterior chamber of the right eye was cannulated with a 30-G needle connected to a reservoir containing Hanks' balanced salt solution. Retinal ischemia was induced by raising the reservoir, thereby increasing the IOP to 120 mm Hg.^2^ The increased IOP, reaching its maximum in 2 min, was maintained for 1 h. At this IOP, systolic collapse of the central artery was confirmed by direct ophthalmoscopy. During the experiment, the animals were kept normothermic with heated jackets. IOP was measured by Tono-Pen XL (Mentor, Santa Barbara, CA, USA) every 10 min during the experiments.

#### Immunohistofluorescence assay

Retinal slides were fixed with 4% paraformaldehyde at room temperature for 20 min and subsequently incubated with 0.5% Triton X-100 for 10 min. Then, the samples were treated with a blocking solution (5% normal goat serum and 2% bovine serum albumin in PBS) for 30 min to prevent nonspecific antibody–antigen binding. *γ*-H2AX, ligase IV, Nrf-1, CREB1 and P-CREB1 expression was detected using the antibody of *γ*-H2AX (Millipore), ligase IV (Santa Cruz), CREB1 (CST), P-CREB1 (CST) and Nrf-1 (Santa Cruz). For the fluorescence visualization of antibody reactions, the primary antibodies were detected using secondary antibodies labeled with the fluorochromes Alexa Fluor 555 or 488 (CST), while the nuclei were detected with DAPI.

### ERG

After overnight dark adaptation, the rats were weighed and anesthetized under dim red illumination and their pupils were dilated with 0.5% tropicamide and 0.5% phenylephrine. The rats were placed on a heating platform to maintain the body temperature at 37 °C, and local anesthetizing eye drops and hydroxypropyl methylcellulose were also applied to the corneal surface. Stainless-steel circellus record electrodes were placed on the central cornea surface. The reference electrode was placed under the tongue and the ground electrode was placed subcutaneously in the tail. Electrode impedance was checked before and after each measurement and was <5 kΩ at 25 Hz. Eyes were stimulated with 2.5 cd•s/m^2^ intensity levels of a light flash delivered by a photic stimulator (Ganzfeld Q400; Roland, Heidelberg, Germany) and the preamplifier bandwidth was set at 0.3–300 Hz. ERG wavelets of each rat were recorded three times and the average amplitudes of a- and b-waves were analyzed. The amplitude of the a-wave was measured as the distance from the baseline to the trough of the a-wave, whereas that of the b-wave was measured as the distance from the trough of the a-wave to the peak of the b-wave.

### NHEJ assay *in vivo*

#### Preparation of plasmid/jetPEI complex

The plasmid pEGFP-N1 was digested with *Eco*R1 and then the concentration was adjusted with ultrapure water to 4 *μ*g/*μ*l. The linearized pEGFP-N1 was mixed with jetPEI (*in vivo*-jetPEI; Polyplus Transfection, Illkirch, France) according to the manufacturer's instructions. In this study, the complexes were prepared as follows: the required amounts of plasmid and transfection reagent were each separately dilute in 10 *μ*l10/% glucose. A volume of 0.05 *μ*l of jetPEI was used per *μ*g of plasmid. The plasmid solution was added to the transfection reagent solution and left for 15 min at room temperature before injection. Plasmid pcDNA3.1 was used as the negative control.

#### Intravitreal injections

A total of 32 adult Sprague–Dawley rats weighing 250–300 g were used for intravitreal injections. Experiments were performed in two groups (16 rats for each group): the experimental group and the control group. The experimental group received subcutaneous injection of lithium chloride (Sigma) once a day, starting from 1 week before ischemia and ending on the day of tissue sampling, whereas the control group received the same amount of PBS only. Daily dose of lithium chloride was 1.0 mEq/kg. Each group was divided into two subgroups (eight rats for each subgroup): I/R experiment was carried out in one subgroup, and the other subgroup with no I/R was treated as the control. At 24 h after I/R surgery, intravitreal injections were put into execution in the right eye of all rats. A volume of 2.5 *μ*l plasmid/jetPEI complex was used for each injection. Intravitreal injections were performed under aseptic conditions using a Hamilton syringe.

#### NHEJ assay

At 48 h after intravitreal injections, the right eyeballs of all rats were enucleated, then were processed to OCT-embedded sections and sequential meridian sections (5 *μ*m thick) were made through the area of the injection hole. The nuclei were stained by DAPI, the positive cells with green fluorescence were counted under the fluorescence microscope (Carl Zeiss).

### Statistical analysis

All *in vitro* experiments were performed in triplicate. Data are expressed as means±SEM. The differences between mean values were evaluated with the two-tailed Student's *t*-test (for two groups) and the analysis of variance (for >2 groups). All calculations and statistical tests were performed by the computer programs Microsoft Excel 2003 (Microsoft, Redmond, WA, USA) or SPSS 11.5 (SPSS, Chicago, IL, USA). *P*<0.05 was considered significant for all analyses.

## Abbreviations

ATF: activating transcription factor
DSBs: double-strand breaks
ERG: electroretinogram
GCL: ganglion cell layer
HR: homologous recombination
INL: inner nuclear layer
I/R: ischemia–reperfusion
NHEJ: nonhomologous end-joining
Nrf-1: nuclear respiratory factor 1
P-CREB1: phosphorylated cyclic AMP-response element binding protein-1
ONL: outer nuclear layer
