# Supplementary material for: Lithium promotes DNA stability and survival of ischemic retinal neurocytes by upregulating DNA ligase IV
Source: Cell Death Dis. 2016 Nov 17;7(11):e2473–. doi: 10.1038/cddis.2016.341 (PMC5260892; doi:10.1038/cddis.2016.341)
Supplement: Supplementary Information [file cddis2016341x1.pdf]

Supplementary Figure S1

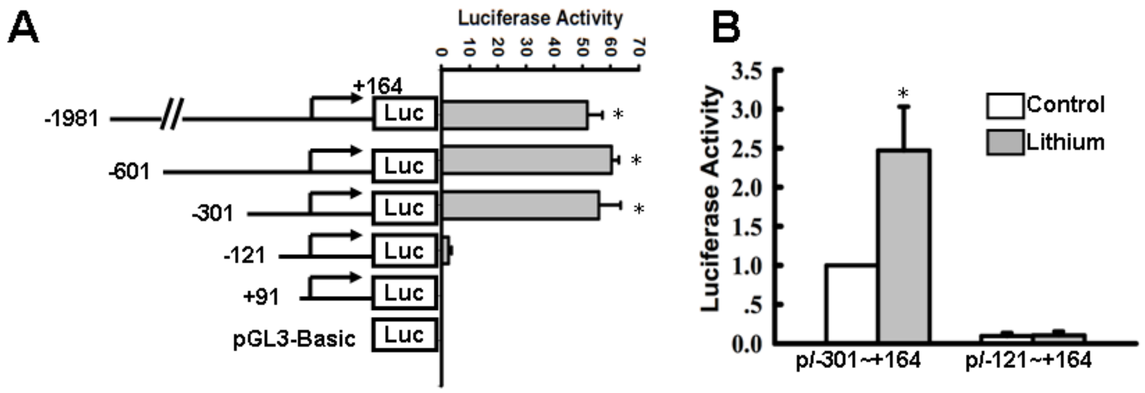

Supplementary Figure S1. The full length sequence of ligase IV promoter is confirmed in retinal neural cells. (**A**) The structure of Ligase IV 5′-end promoter constructs. Ligase IV promoter fragments of different lengths but with the same 3′-end were cloned into vector PGL3-Basic. Glioma C6 cells were co-transfected with reporter plasmids and pRL-CMV, and after twenty-four luciferase activity was assayed. (Luciferase levels were normalized with a Renilla luciferase) Here, expression from the promoter encompassing -301 to +164 was over 10-fold more active than the construct containing the fragment of -121 to + 164 (**B**) Primary retinal neurocytes following vehicle- or lithium-treatment, in serum-free medium, co-transfected with reporter plasmids (p-301 to +164 or p-121+164) and pRL-CMV for twenty-four hours. The levels of luciferase activity were normalized to Renilla luciferase activity. Lithium treatment significantly increased the inducibility of the construct (from -301 to +164), but had no effect on the p-121+164 construct. n = 3 for each group, *P < 0.05.

Supplementary Figure S2

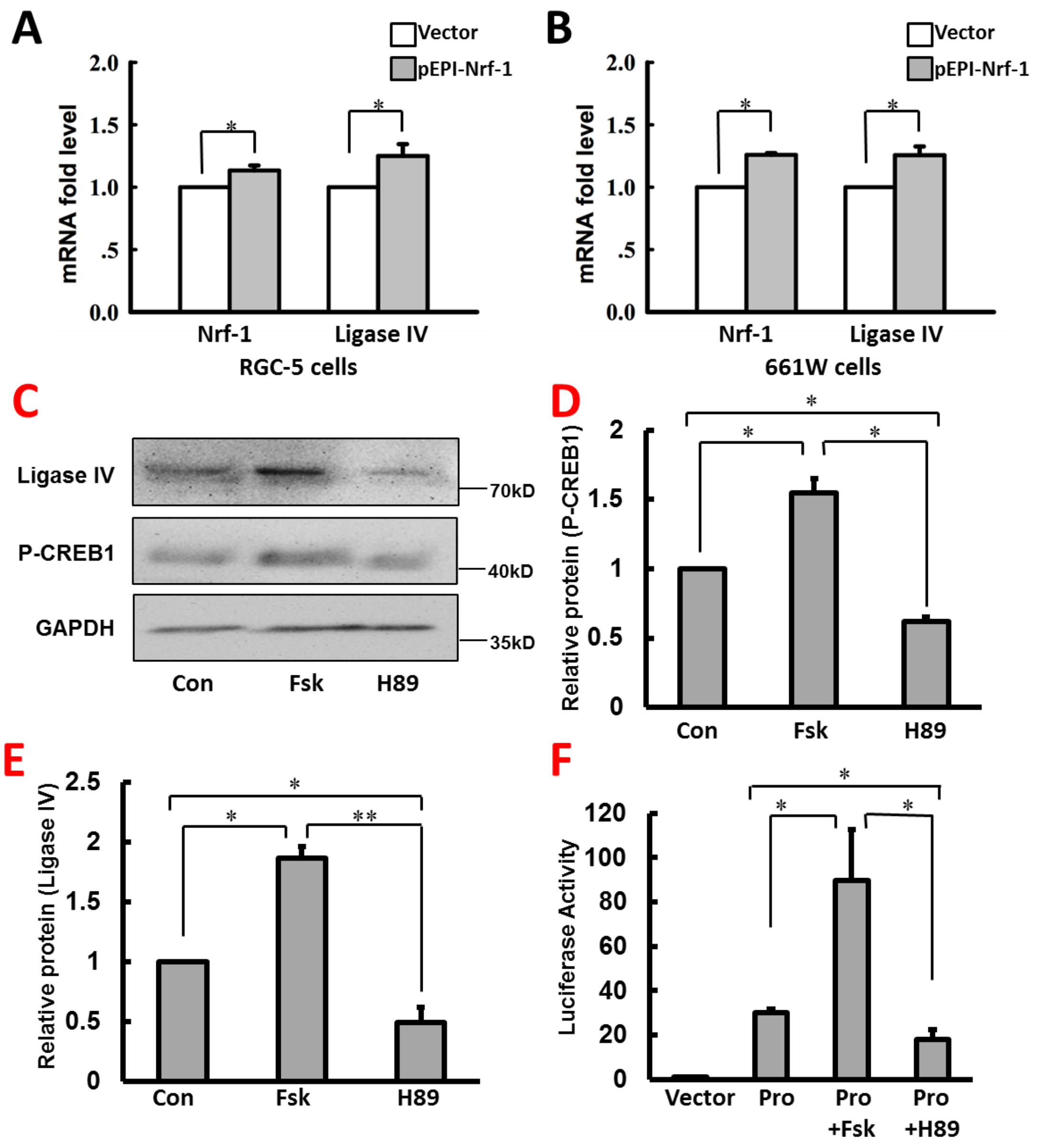

Supplementary Figure S2. Nrf-1 and P-CREB1 are confirmed in regulating the transcription of Ligase IV. Over-expression of Nrf-1 promotes transcription of Ligase IV in retinal neurocytes. RGC-5 (**A**) and 661W (**B**) cells transfected with the plasmid, pEPI-Nrf-1 and empty vector, respectively. Real time RT-PCR assay demonstrates that Nrf-1 overexpression (RGC-5, Con, 1±0; Con+pEPI-Nrf-1, 1.13±0.07; 661w, Con, 1±0; Con+pEPI-Nrf-1, 1.17±0.03) notably upregulated Ligase IV expression (RGC-5, Con, 1±0; Con+pEPI-Nrf-1, 1.25±0.12; 661w, Con, 1±0; Con+pEPI-Nrf-1, 1.20±0.10) in retinal neurocytes cultured in full medium n = 3 for each group, *p<0.05. (**C**) Western blot analysis

indicates that CREB1 phosphorylation and Ligase IV in retinal neural cells is up-regulated by treated with PKA activators (Forskolin) (10μM) and down-regulated by treated with PKA inhibitors (H89) (10μM). (**D**) The relative CREB1 phosphorylation in retinal neurocytes was quantified by densitometry. PKA activators (Forskolin) notably enhance CREB1 phosphorylation and PKA inhibitors (H89) significantly decrease CREB1 phosphorylation in retinal neurocytes. (Con, 1; Fsk, 1.55 ±0.1; H89, 0.62 ±0.03) n = 3 for each group, *$p<0.05$. All error bars represent SEM. (**E**) The relative expression of ligase IV in retinal neurocytes was quantified by densitometry. The expression of Ligase IV is up-regulated after treated with PKA activators (Forskolin) and down-regulated after treated with PKA inhibitors (H89) (Con, 1; Fsk, 1.86 ±0.1; H89, 0.49 ±0.13) n = 3 for each group, *$p<0.05$, **$p<0.001$. (**F**) Primary retinal neurocytes were co-transfected with reporter plasmids (Pro) and pRL-CMV for twenty-four hours, the retinal neurocytes were incubated with PKA activators (Forskolin) or PKA inhibitors (H89) respectively at the last four hours. The levels of luciferase activity were normalized to Renilla luciferase activity. The results showed that the activity of Ligase IV promoter was enhanced by PKA activators (Forskolin) and inhibited by PKA inhibitors (H89) retinal neurocytes (vector, 1; Pro, 30.1 ±1.5; Pro+Fsk, 89.5 ±23; Pro+H89, 18.2 ±4.3) n = 3 for each group, *$p<0.05$. All error bars represent SEM.

Supplementary Figure S3

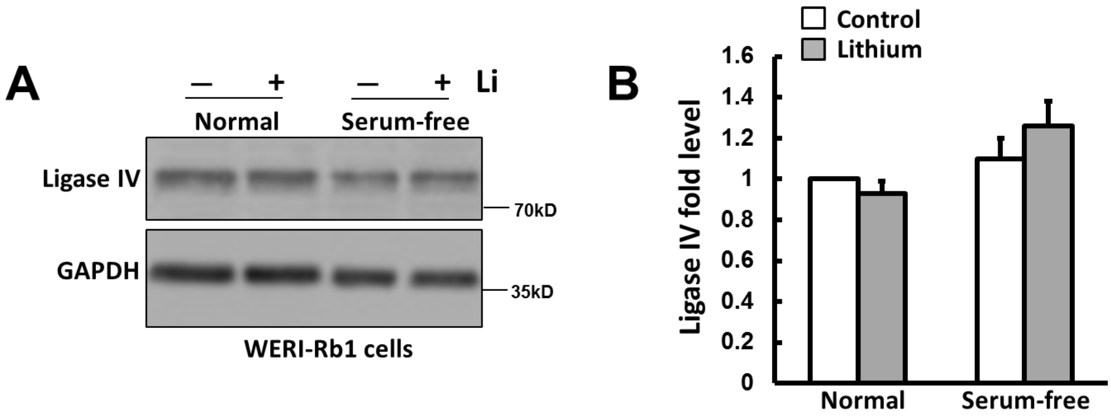

Supplementary Figure S3. The expression of DNA Ligase IV in retinal neurocytes and WERI-Rb1 cells. (**A**) Western blot analysis indicates that Lithium does not affect Ligase IV expression in full medium cultured or serum-starved WERI-Rb1 cells. (**B**) The relative expression of ligase IV in WERI-Rb1 cells was quantified by densitometry. Nutritional starvation does not significantly alter the expression of Ligase IV in lithium-treated WERI-Rb1 cells (Con, 1±0; Con+Li, 0.93±0.06; Serum-free, 1.1±0.1; Serum-free+Li, 1.26±0.12). n = 3 for each group.

Supplementary Figure S4

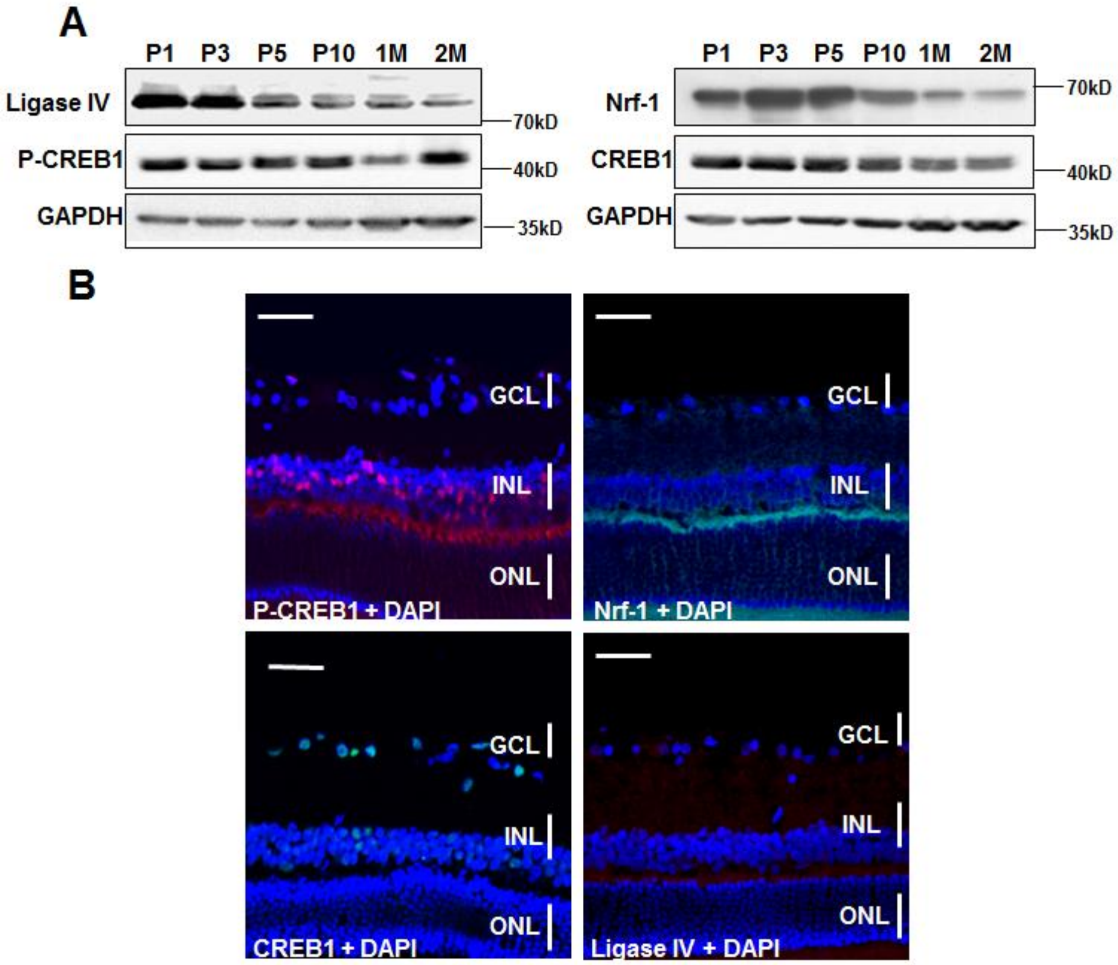

Supplementary Figure S4. Retinal expression pattern of Nrf-1, P-CREB1, CREB1 and Ligase IV in the development of SD rat retina and the adult rat retina are stained by immunofluorescence. (**A**) Expression level Nrf-1 and ligase IV is developmentally down-regulated in the rat retina, whereas, that of P-CREB1 is constant. GAPDH was included as a loading control. (**B**) Immunofluorescence staining of Ligase IV (red), P-CREB1 (red), CREB1 (green) and Nrf-1 (green) in adult rat retina, which is consistent with the results of western blot above. Scale bars: 50 µm.

Supplementary Figure S5

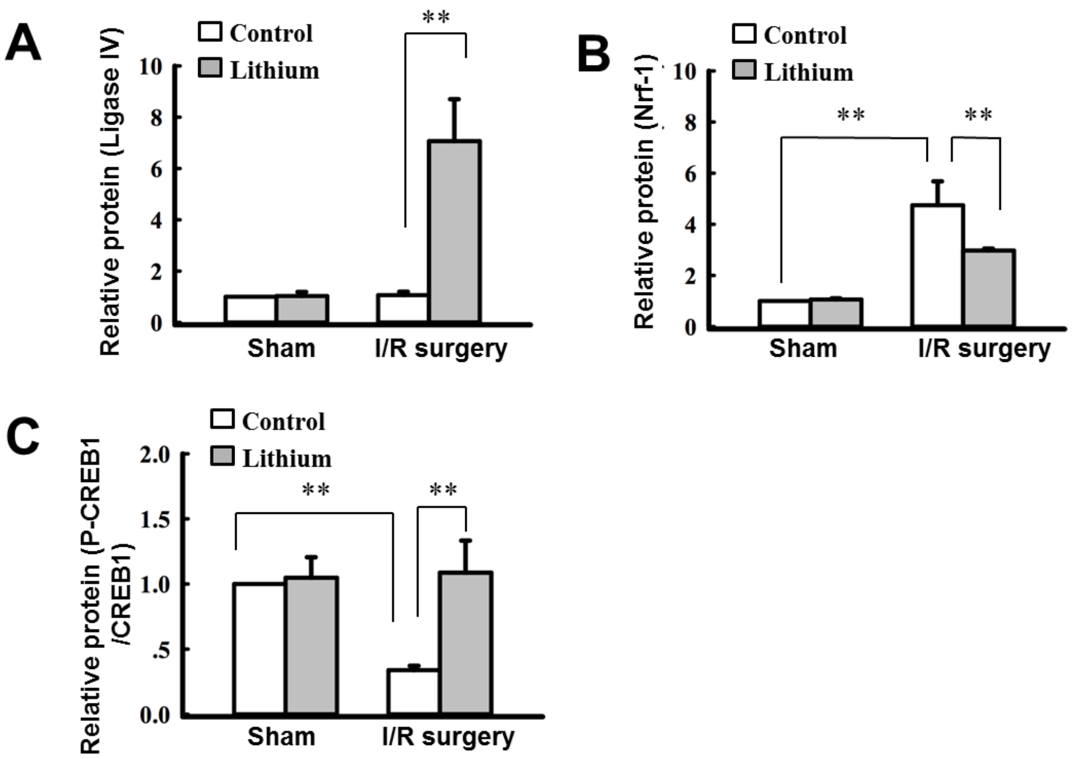

Supplementary Figure S5. Relative levels of Ligase IV, Nrf-1, and P-CREB1/CREB1 (ratio to GAPDH) on the Western blots (Fig. 5B) in retinal tissue were presented. Lithium does not affect the expression of Ligase IV, Nrf-1 and P-CREB1/CREB1 in retina without I/R surgery (**A**) (**B**) (**C**). I/R surgery significantly down-regulates CREB1 phosphorylation /CREB1 in retina (**C**), but increases Nrf-1 (**B**). In contrast, in lithium-medicated retina, I/R surgery increases P-CREB1/CREB1 (**C**), and mildly decreases Nrf-1 (**B**). Correspondingly, lithium treatment up-regulated Ligase IV expression in retina after following I/R surgery (**A**). n = 3 for each group, $^{**}p<0.001$. All error bars represent SEM.

Supplementary Figure S6

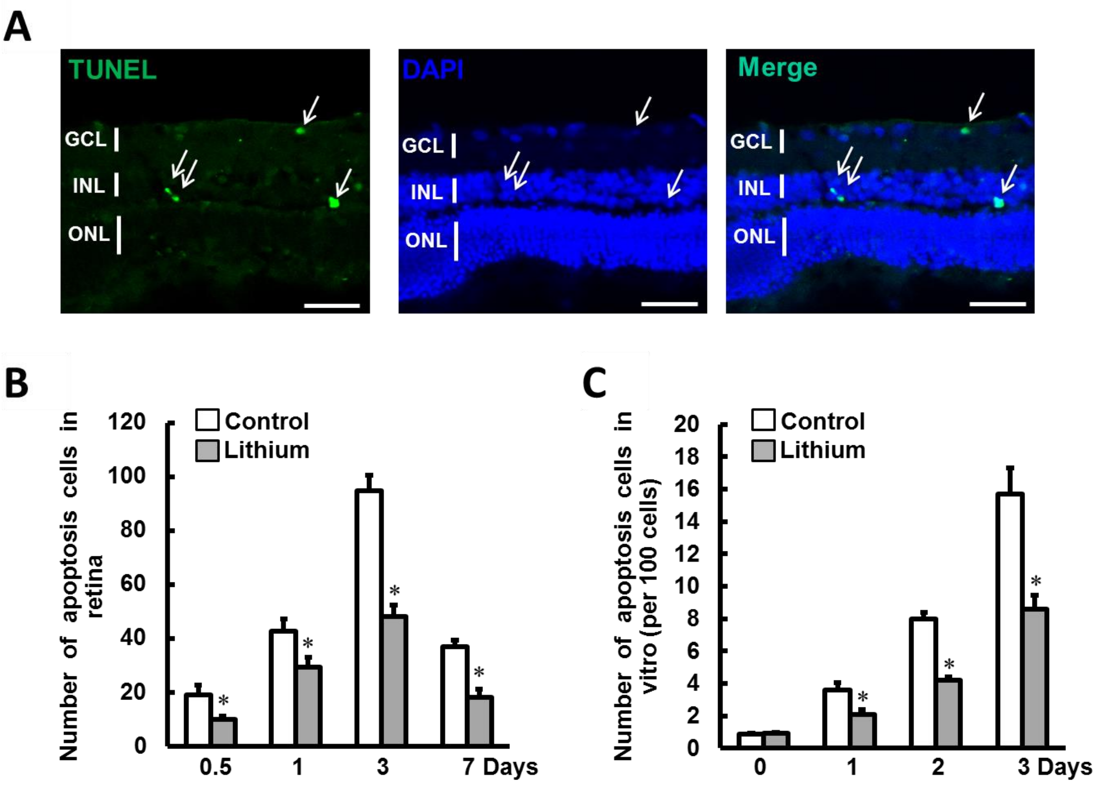

Supplementary Figure S6. Lithium treatment reduces the number of apoptosis cells in retina after I/R surgery and in retinal neurocytes cultured in serum-free medium. (**A**) The number of apoptosis cells in retina was calculated at 0.5, 1, 3 and 7 days after I/R surgery by TUNEL assay. Scale bars: 50 µm. (**B**) The number of apoptosis cells was significantly decreased in retina pre-treated with Lithium (0.5d, 19±3.7; 1d, 42.7±4.5; 3d, 94.7±5.7; 7d, 37±2.4) compared with control (0.5d, 10±1.4; 1d, 29.3±3.7; 3d, 48.3±4.1; 7d, 18.3±3.1) at different time points after I/R surgery. n = 3 for each group, *$p<0.05$. (**C**) The number of apoptosis cells (per 100 cells) was notably decreased in retinal neurocytes pre-treated with Lithium (1d, 3.6±0.43; 2d, 8±0.41; 3d, 15.7±1.59) compared with control (1d, 2.1±0.28; 2d, 4.2±0.21; 7d, 8.6±0.87) at different time points after the cells cultured in serum-free medium. n = 3 for each group, *$p<0.05$.
